# “I would rather do it myself”: injection initiation and current injection patterns among women who inject drugs in Tijuana, Mexico

**DOI:** 10.1186/s12954-021-00554-9

**Published:** 2021-10-13

**Authors:** Allison Stewart, Brooke S. West, Claudia Rafful, Kenya Lazos, Jennifer Jain, Patricia Gonzalez-Zuniga, Teresita Rocha-Jimenez

**Affiliations:** 1 Columbia University Mailman School of Public Health, New York, NY USA; 2 Columbia University School of Social Work, New York, NY USA; 3 Faculty of Psychology, National Autonomous University of Mexico, Mexico City, Mexico; 4 Center on Global Mental Health Research, National Institute of Psychiatry, Mexico City, Mexico; 5 Wound Clinic Tijuana, Tijuana, Mexico; 6 Division of Infectious Diseases of Global Public Health, School of Medicine, University of California, La Jolla, CA USA; 7 Society and Health Research Center, Faculty of Humanities, Universidad Mayor, Santiago de Chile, Chile

**Keywords:** Injection drug use, Gender, Risk environment, Women who inject drugs, Harm reduction, USA–Mexico border

## Abstract

**Background:**

Women who inject drugs (WWID) experience unique risks and adverse health outcomes related to injection initiation and patterns of injection drug use. However, there is limited information on injection initiation experiences and injection patterns among women and the protective strategies employed to limit injection-related harms, especially in low- and middle-income settings. Therefore, this study sought to explore injection initiation and current injection patterns (e.g., relying on someone else to inject) among women who inject drugs and engage in sex work in Tijuana, Mexico.

**Methods:**

Semistructured in-depth interviews were conducted with 30 WWID on the following topics: injection initiation, current injection patterns, places where women inject, and protective strategies (i.e., risk reduction). All interviews were audio-recorded, transcribed, and de-identified. An inductive thematic analysis was conducted to identify and compare common themes and patterns across participants.

**Results:**

The interviews revealed that the vast majority of study participants were first initiated by another person who injects drugs (PWID), often a male sexual partner. However, the majority of the women transitioned to become self-injectors in order to avoid risks associated with relying on others for injection, including overdose, interpersonal violence, sexual abuse, and wounds. Those who relied on others indicated that they would prefer to inject themselves without assistance from others if they were able to.

**Conclusions:**

The narratives uncovered in this study reveal the importance of multiple risk environments in shaping perceived risks associated with injection drug use among women in Tijuana, Mexico. Specifically, the interviews elucidate the connection between interpersonal relationships with other PWID and protective strategies used to minimize risk and harm. These findings highlight the need for women-centered harm reduction programs to facilitate the development of safer drug use environments among WWID in Tijuana, Mexico.

## Background

Although women who inject drugs (WWID) comprise only 20% of all people who inject drugs (PWID) globally, they face a myriad of gender-specific risks including intimate partner violence, intersections between sex work and drug use, and social and economic marginalization [1–3]. These factors may contribute to injection initiation, increased risk from injection patterns, and other adverse health outcomes associated with injection drug use [2, 4–6].

Compared to men who inject drugs (MWID), WWID are more likely to experience injection-related problems (e.g., poor injecting practices contributing to increased risk of transmission of blood-borne diseases) and faster progression from first use to substance use disorders. Additionally, prior research has shown that WWID tend to be at higher risk of acquiring HIV, other sexually transmitted infection (STIs), and hepatitis C compared to MWID. These gender disparities may be attributed to the overlap between drug use and sex work and a reliance on male partners for drug acquisition, preparation, and injection [6–8]. Rates of gender-based and intimate partner violence have additionally been found to be two to five times higher among WWID compared to women who do not use drugs [9]; such experiences place women at greater risk for HIV/STI and other health-related harms.

Previous research indicates that gender influences injection initiation within spatial, social, and economic risk environments; initiation processes therefore may vary depending on gender and geo-cultural location [10]**.** According to previous research, women are more likely than men to be injected by someone else their first time, often a sexual or romantic partner [4, 5]. Women are more likely to receive initiation assistance in initiation by a male sexual partner, while MWID are more likely to be assisted by a casual acquaintance [10–13]. Additionally, men in Tijuana are twice as likely to provide injection initiation assistance when compared to women [14].

It has also been found that there is a greater overlap between sexual and injection networks among WWID compared to MWID and women’s efforts to maintain drug use are often linked to sustaining daily life, such as obtaining food, housing, and other necessities [5].Women are also more likely to continue to be injected by another PWID and are more susceptible to the risks associated with being injected by someone else [4–6].

Other studies have concluded that many women are self-injectors and that many receive their first injection from a female, rather than a male, partner [15]. Another study conducted among PWID in Vancouver discovered that women were twice as likely as men to report requiring assistance with injecting [10, 16]. Although there is a growing body of research covering injection drug use patterns among WWID, results thus far are mixed and inconclusive, indicating that initiation process and drug use patterns among PWID may vary depending on geographic context and associated risk environments [10].

Patterns of injection drug use and associated health outcomes among WWID are of particular concern in Tijuana, Mexico, a border city in Northern Mexico and a central node along a major drug trafficking route, given its proximity with the USA [17]. Cross-border mobility resulting from work, tourism, migration and deportation, and drug trafficking has contributed to a particularly high incidence of HIV and other health risks compared to other regions of Mexico [18–20]. The HIV prevalence in Tijuana is additionally concentrated in sub-epidemics among key populations including PWID, sex workers, and men who have sex with men [21]. A confluence of dynamic factors in Tijuana, including population mobility, poverty, high availability of illicit drugs, and changing drug and border enforcement policies create an environment that is conducive to HIV transmission and that place PWID in positions of structural vulnerability [20, 22, 23].

Tijuana is one of the most violent cities in Mexico, and this violence has a particular impact on women. In 2016, Tijuana had the second highest rate of feminicides in the country (7.2–13 per 100,000 women) [24]. A recently published book analyzes the violence against women in Tijuana as an epidemic and highlights the intersection between drug trafficking, drug use, and the role of the border [25].

Physical placement along a major drug trafficking route combined with micro-level exposure to ample drugs, violence, punitive policing practices, and engagement in sex work place women in Tijuana in precarious conditions with limited interventions [10, 23]. Previous studies conducted in Tijuana that examine risk environments among women who inject drugs suggest that there is a complex intersection between social or structural factors and gender power dynamics that impact women’s lives and potentially their injection initiation and injection patterns [10, 14, 26].

The interplay of diverse individual, social, and structural factors that contribute to health risks faced by WWID can be depicted by the risk environment framework, which explains how micro- and macro-level influences shape individual-level risk behavior in four different risk environments: physical, social, economic, and policy. Thus, the risk environment framework shifts the responsibility of harm away from the individual and toward a broader social and structural context. The risk environment framework has important implications for harm reduction practices as it emphasizes the importance of addressing social contexts and recognizes the potential of non-health-oriented interventions [27, 28].

Although recent studies have begun to document the unique patterns of injection drug use among women, there are lingering gaps in understandings around the specific experiences and needs of women who inject drugs in Tijuana, Mexico, contributing to challenges in developing policy and appropriate interventions. This study, therefore, aims to utilize the narratives of women in Tijuana to enhance understanding of risks and protective factors associated with varying patterns of injection drug use and ultimately inform harm reduction interventions catered to women in Tijuana.

## Methods

The in-depth interviews for this analysis were conducted with 30 female sex workers who inject drugs (FSWID) as part of the mixed-methods *Intersecciones* study (PI: West, K01DA041233), which aimed to examine the spaces in which women inject drugs and trade sex, including venue network affiliations, and the manner in which the intersection of place and network characteristics drive HIV/STI transmission. Study participants were recruited from the *Proyecto El Cuete IV* cohort (PI: Strathdee, R01DA037773), a longitudinal study of drug policy and HIV among people who inject drugs in Tijuana, Mexico. The *Proyecto El Cuete IV* cohort study methods have been previously described in full [29]. Participants for the in-depth interviews were selected using a purposive sampling scheme that aimed to capture a diversity of experiences and perspectives of injection initiation and injection patterns. To be eligible for participation in the present study, women had to be at least 18 years old, report injection drug use in the past 6 months, report exchanging sex for money, drugs, or other goods in the past 6 months, report sharing injection equipment or sex without a condom in the past 6 months, and speak English or Spanish [29].

### Data collection

Semistructured in-depth interviews with FSWID in Tijuana, Mexico, were conducted between September and October 2019. Qualitative data collection was led by TR, and field staff from *El Cuete* project. Upon written informed consent, in-depth interviews were conducted, lasted between 60 and 90 min, and were audio-recorded. The semistructured interview guide was developed by BW, TR and CR based on previous field work and literature review and included questions about injection initiation, current injection patterns, places where women inject, and protective strategies. Extensive field notes of each participant and how they were invited to participate were also taken. One participant declined to be audio-recorded, but as she still wanted to participate, detailed field-notes of that interview were taken and analyzed. Participants received $20 USD as compensation for their time. All study procedures were approved by the IRBs at Columbia University and the University of Xochicalco, in Tijuana, Mexico.

### Data analysis

All interviews were transcribed and translated by trained, bilingual staff. Any personal identifiers were removed, and each participant was identified by a unique pseudonym. A codebook was created that was modified iteratively as the analysis progressed. Family themes were created, followed by subthemes, and support codes to provide more details of the diverse experiences reported. Support from the field staff was provided to ensure certain experiences were interpreted accurately. The online software Dedoose 8.2.14 was used to manage data coding. Interviews and fieldnotes were analyzed to understand participants’ injection initiation experiences and current injection patterns. An inductive thematic analysis approach was utilized to identify and compare common themes and patterns across participants [30, 31].

Interview findings were organized by participants’ injection experiences (Table 1). Specific categorizations included factors influencing their injection initiation experiences (e.g., the influence of PWID or impactful life events), patterns of self-injection and helping others to inject, and patterns of relying on other people to inject. The risk environment framework was utilized to guide the analysis.

**Table 1:** Injection drug use patterns and among women who inject drugs in Tijuana, Mexico, 2019 (N = 30)

Experience	Description	*n (%)*
***Injection initiation***		
Influenced by others	Injection initiation was influenced by someone else, either by direct injection or exposure to others’ drug use	21 (70.0)
Not influenced by others	Injection initiation was not influenced by anyone else. Other factors such as the use of other drugs or key life events may instead play a larger role	9 (30.0)
Use of other drugs	Previous use of other drugs was mentioned in relation to the participants’ experience of beginning to inject drugs	10 (33.3)
Influenced by key life event/experience	Key life events or experiences, such as deportation, medical concerns, or the death of a family member, contributed to the participants’ initiation into injection drug use	10 (33.3)
Learned to inject by watching others	In order to learn to inject themselves, the participants watched and learned others’ injection techniques	14 (46.7)
***Participants who know how to inject themselves***		
Inject themselves and are street-hit doctors	The participant describes themselves as a “street-hit doctor” (i.e., someone who assists others with injection on a regular basis, usually in exchange for money, drugs, or other compensation)	1 (3.3)
Inject themselves and occasionally help others	The participant helps others to inject, sometimes for compensation such as drugs or money	16 (53.3)
Inject themselves but do not help others	The participant injects themselves but not does help others to inject	7 (23.3)
Never have initiated someone	Of those that know how to inject themselves and others, the participant has never helped someone else to inject for the first time	12 (40.0)
Have initiated someone	The participant has helped someone else to inject for the first time	4 (13.3)
***Participants who rely/get help from someone to inject***		
Have someone close and whom they trust to help them	The participant gets help from someone close to them, whom they trust, such as a friend, roommate, or intimate partner helps them to inject	10 (33.3)
Asks other PWID	The participant receives help from other PWID, for example, acquaintances but not necessarily someone they know well or trust	8 (26.7)
Ask anyone who is around	The participant asks for help from anyone who is around and might not know to inject drugs	1 (3.3)
Knows how to inject themselves but currently needs help occasionally	The participant knows how to inject and may have in the past injected themselves, but they currently rely on others for help injecting due to difficulties finding their veins, fear of getting wounds, or other health concerns	4 (13.3)
Relies on others to inject them but they help others	The participant relies on someone else to help them inject, often due to difficulties finding their own veins, and they also help others inject drugs	2 (6.7)

## Results

### Participant characteristics

Among a total of 30 participants, 27 (90%) considered themselves to be Hispanic, Latino, or Mexican. The majority of participants (73%) were married, and only four (13.3%) were single or never married. Almost one-half of participants (43.3%) had completed preparatory school or a higher level of schooling. The majority of participants (76.7%) were in Mexico when they first injected drugs, and the mean age of first injection was 23.48 with a range from 12 to 45 years old.

## Summary of findings

In-depth interviews revealed a diversity of patterns with respect to two main categories of experiences: (1) injection initiation and pathways to current injection drug use practices, and (2) current injection patterns. Injection initiation and current injection experiences (e.g., self-injection, relying on others, injecting others) were influenced by other PWID, by key life experiences, such as migration, economic factors, medical issues, or previous use of other drugs; each woman’s individual experience was shaped by various contextual factors. Injection patterns (e.g., where and with whom the women injected drugs) were commonly connected to perceived risks and often changed during the course of their drug use trajectory.

### Injection initiation and pathways to current drug use practices

Micro-level risk environments encompassing the participants’ relationships with other PWID were a central element of their injection initiation experiences; they generally described the first time they injected heroin to be influenced by or associated with another person. Only two out of thirty participants injected themselves for the first time, and only two were initiated by a stranger. More commonly, the women recounted experiences of someone close to them, such as a romantic partner, friend, or family member, initiating them into injection drug use.

“*He [uncle] told me that with the heroin, he didn’t feel a thing and that he felt really good and all of his problems went away and then he asked me if I wanted some,”* reported one participant as she recounted the influence of her uncle’s injection drug use *(Rosa, age 35 when interviewed, age 12 at initiation).*

One participant described pressure from her friends to initiate injection drug use: *“I didn’t want to try it, but you know how friends are and I had to try it. They told me that nothing bad would happen” (Daniela, age 29 when interviewed, age 20 at initiation).*

Several participants were not only influenced by other PWID to initiate injection drug use but were also directly injected by another person their first time. Almost two-thirds of participants (*n* = 19) reported that they were first injected by a man who injects drugs, often in the context of an intimate relationship.

*“He [romantic partner] injected himself and then he injected me so that I could try it and that’s when I liked how it felt and that’s how I started using heroin and I couldn’t stop” (Luisa, age 25 when interviewed, age 16 at initiation).*

*“I moved here with a guy who was seventeen years old. He had already used heroin and was up to no good. He injected me my first time” (Alison, age 33 when interviewed, age 13 at initiation).*

A few of the participants were first injected by a female friend or female partner and a small minority were initiated by a family member.

*“I was so young. I didn’t know what I was doing. I was only 15 years old. And I actually started [to inject] with my mom,”* one participant responded when asked if someone helped her inject for the first time *(Estela, age 47 when interviewed, age 15 at initiation).*

Although uncommon among this study’s sample, a couple participants reported that they were coerced into injection initiation. One participant was unwillingly exposed to heroin by a female friend while she was falling asleep in a drunken state.

*“I was falling asleep and I felt how she put the needle here inside my nose,”* she said *(Genoveva, age 41 when interviewed, age 20 at initiation).*

Another stated, *“The first time that I consumed heroin was without my consent,”* recounting the beginning of her injection drug use in which her partner injected her with heroin to alleviate pain from a gunshot wound *(Vilma, age 41 when interviewed, age 33 at initiation).*

In addition to experiences of direct injection by another person, some of them added that observation of other PWID around them contributed to their initiation of drug injection. Many stated that a partner, friend, or family member was already using injection drugs before their own initiation, sometimes when the participants were underage.

One participant said that *“there were some problems going on in our house and it just caught my attention,”* referring to how her sister’s heroin use incited her own curiosity to initiate injection *(Sofia, age 39 when interviewed, age 23 at initiation).*

Another mentioned, *“My boyfriend used to shoot heroin, so I used to watch him shoot it and I started to get curious about what he felt” (Isabel, age 20 when interviewed, age 16 at initiation).*

Additional influences for injection initiation included key life experiences at both the micro- and macro-level, including medical concerns and issues related to migration and deportation. For example, one woman said:

*“When I came back here, migration* [authorities] *took all of them* [pain pills] *away. I felt really bad because those give you [withdrawal symptoms] as well and I didn’t have any money to go to the doctor, so it was easy for me to buy heroin because it was the closest thing to that” (Jazmin, age 48 when interviewed, age 23 at initiation).*

*“It was because I was in the penitentiary. I did it just to pass the time, so that it could go by faster. I was curious. Everyone inside there was hooked and I was young and stupid” (Lucia, age 35 when interviewed, age 16 at initiation).*

*“I was very disappointed because they had just told me I was being deported for life, but I had not seen a judge or had a hearing of any kind and I felt like I wanted to die because I had a child back in the U.S. who was epileptic with a brain tumor” (Elena, age 56 when interviewed, age 45 at initiation).*

Other participants connected their initiation of injection drug use to previous use of other drugs and economic factors.

*“I started to get high on pills [Oxycodone and Vicodin] and he* [partner] *kept telling me heroin was the same thing because they were opioids. I was doing four pills a day and I couldn’t afford it so that’s why I stopped using them and started using heroin”* one participant explained *(Laura, age 25 when interviewed, age 23 at initiation).*

Although the early experiences of injection drug use for many of the participants involved other people who helped them inject, the majority eventually learned to inject themselves. A small number of participants had prior knowledge of injection and injected themselves without difficulty. For example, one participant knew how to inject others from previous nursing experience. However, most of the participants learned by observing the injection techniques of other PWID.

“I did the same thing that my uncle did the day before and I started injecting myself and I learned very quickly” (Rosa, age 35 when interviewed, age 12 at initiation).

*“*[I learned by] *watching how other people would inject themselves and on which side they did it or if they put it directly on the vein or to the side of it and stuff like that” (Luisa, age 25 when interviewed, age 16 at initiation).*

### Current injection patterns

A variety of experiences of current injection were found among the participants, including exclusive self-injection, exclusive reliance on others, both self-injection and reliance on others, as well as patterns of helping others to inject. The motivation for injecting oneself instead of relying on others for injection was closely tied to perceived risks and intentions to avoid harm. These perceptions were formed by experienced or anticipated vulnerabilities shaped by micro-level social and physical risk environments, such as previous experiences with wounds and overdose.

#### Self-injection

When asked about current injection patterns, the vast majority of the participants (*n* = 24) identified as self-injectors and currently inject themselves. Many of the women saw benefits of self-injecting rather than relying on others, including safety and competency, stemming from previous experiences of theft and abuse, and also fear or intentional overdose or wounds by others. The participants explained that other PWID, usually strangers, would often steal from them, intentionally inject too strong a dose that could result in overdose, or hit the jugular vein, which could cause harm or even death.

“I do it by myself. I don’t like when someone else does it. Because if something goes wrong, I don’t want to blame anyone but myself. I’m also scared because people are careless and nobody will be as careful as you are” (Luisa, age 25 when interviewed, age 16 at initiation).

“It’s always by myself. I just don’t like to be around other people who use. Just the way some of them are. Like I can’t bring them into my apartment for one because they steal. They just lie and steal sometimes” (Estela, age 47 when interviewed, age 15 at initiation).

“I’ve heard stories where people do it on purpose, for example, they shoot you in the wrong vein… you faint and are unconscious, enough time for them to steal your shoes and everything else…Or they can switch the needle and they don’t give you any drugs and you don’t get the good stuff” (Alison, age 33 when interviewed, age 14 at initiation).

Others mentioned that being able to inject themselves enabled them to inject drugs more frequently and not worry about having to find others for immediate assistance, for example if they were experiencing withdrawal symptoms.

“It’s not that easy to find someone to hit you, that you can trust, because people are scandalous” (Laura, age 25 when interviewed, age 23 at initiation).

“When I couldn’t find anyone to help then I would do it myself, although I would struggle a lot. After that, I started paying attention when they would inject me here in my arm and when you have those withdrawal symptoms you think: ‘well, I have to try maybe with some luck I can find the vein’ because those withdrawal symptoms are worse than if you hurt yourself with the needle” (Ines, age 50 when interviewed, age 20 at initiation).

Finally, some of the participants explained the benefits of having privacy while injecting and avoiding issues caused by sharing drugs with other PWID.

*“I don’t like people watching me do it. I’ve helped other people get injected, but I don’t like them watching me, I get embarrassed,”* someone explained *(Daniela, age 29 when interviewed, age 20 at initiation).*

*“*[Injecting with others] *creates issues. Because I have one more line than you, then you start complaining and that creates issues and has even caused death just because someone has more heroin. There are many things that can create problems so that’s why I think it’s better to do it myself” (Mireya, age 60 when interviewed, age 15 at initiation).*

Despite the benefits explained above, there are challenges to self-injection, including the challenge of findings one’s veins and injecting safely. The participants often suffered from wounds, abscesses, and infections while learning to self-inject.

*“I learned how to inject myself, but I hurt myself and now I my arm is very swollen and hurt because I hit a nerve. My husband always injects me but this time I injected myself and I hurt myself” (Claudia, age 54 when interviewed, age 42 at initiation).*

*“We addicts say that there is an opposite vein. It’s a vein that has pure blood rushing through it, but we call that vein the opposite vein because if we inject that vein on our neck, we risk getting overdosed or killed. You have to know where you can inject*” (Luisa, age 25 when interviewed, at 15 at initiation).

Some participants never learned to inject themselves due to problems finding their veins and therefore an inability to inject themselves properly. Another participant mentioned that she did not want to learn how to inject because she believed she would therefore be less likely to develop a substance use disorder.

*“I would tell him* (partner) *that I didn’t want to learn how to inject because that way it would be easier for me to quit. Whenever he ended up in* [local jail] *I had to go to the streets looking for someone to inject me because I couldn’t do it by myself” (Elena, age 56 when interviewed, age 45 at initiation).*

One participant additionally mentioned the challenge of having to buy drugs for oneself instead of splitting the cost with others.

*“If I have the money, I will buy it by myself but if I don’t have enough, I will share it with someone, so we buy it half and half” (Mireya, age 60 when interviewed, age 15 at initiation).*

#### Relying on others

Although many of the participants reported that they injected themselves, the vast majority (83%) also stated they relied on others to help them inject, either exclusively or only occasionally when needed. This was most often someone whom they trusted, such as a friend, roommate, or partner. However, some participants asked other PWID, not necessarily someone they trusted, for assistance, and one participant reported that she asked anyone who was around that might know how to inject.

*“I live with someone who uses heroin as well and he is the one who injects me most of the time and we share the same drugs but not the same needle” (Elena, age 56 when interviewed, age 45 at initiation).*

*“I still don’t know how to inject myself that well. I need help every time that I want to get a fix. I struggle with finding my vein so I’m always looking for someone or paying someone to help me” (Mercedes, age 37 when interviewed, age 29 at initiation).*

*“It was always the same people, I never asked other people, it was always the same. It was like I had my own “doctor*” [i.e., a fellow PWID and that assists others in drug injection] *who always treated me” (Ines, age 50 when interviewed, age 20 at initiation).*

Importantly, many of the women explained that asking for help with injection brought additional risks, such as intentional wounds or overdose, theft, and abuse, as stated earlier.

*“I’m scared of getting HIV because what they do is that they rush you when they are going to inject you and they tell you to hurry up and before they inject you, they switch the needle and give you that fake heroin and that’s how they steal the heroin from you and that’s how you can get AIDS” (Elena, age 56 when interviewed, age 45 at initiation).*^1^

It was found that some of the participants continued to rely on men over time and some said they felt pressure to be in a relationship with a man in order to have reliable assistance with injection.

*“I needed to go with him so he could inject me, and I had to share some of my* [heroin]*,”* one participant stated *(Isela, age 38 when interviewed, age 18 at initiation).*

*“We are more vulnerable, and we are at the mercy of men because men have the means to defend themselves,”* one woman said as she expressed the vulnerability attached to relying on men *(Lucia, age 35 when interviewed, age 16 at initiation).*

Overwhelmingly, those that relied on others for injection reported that they would prefer to inject themselves if they were able to.

#### Injecting others

More than half of the women additionally helped other PWID to inject. Some helped others only occasionally, while some participants injected a partner or friend on a regular basis. It was commonly reported as a reciprocal exchange, sometimes involving the exchange of drugs or money, but at other times, for no compensation at all. One participant considered herself to be a “street-hit doctor,” frequently helping other PWID to inject, in exchange for drugs or money.

“I’ve always helped other people with it. That’s what I do for money, I’m what they call a street doctor,” the participant stated (Carmen, age 49 when interviewed, age 39 at initiation).

Some women specifically stated that they did not help others to inject, despite knowing how to, typically for fear of contributing to wounds or overdose. Most said they only helped friends or acquaintances, those they can trust have used heroin in the past. One woman explained that she refused to help people who she didn’t know beforehand.

“I don’t know if they are already heroin users or if it’s their first time and I don’t want to kill anyone,” she said (Sofia, age 39 when interviewed, age 23 at initiation).

When asked if they had ever initiated anyone into injection drug use, only four participants reported that they had. Most expressed that they refuse to initiate others, citing their disapproval of their own injection practices.

*“A young guy asked me once, but I didn’t it. Because the guilt would be too much after that,”* one woman expressed *(Luisa, age 25 when interviewed, age 16 at initiation).*

Another woman said, *“I cannot destroy the life of a person,”* while another mentioned, *“us addicts are dead people in live bodies,”* both expressing dissatisfaction with their current life and refusal to initiate others into drug-related problems *(Elena, age 56 when interviewed, age 45 at initiation).*

#### Injecting with others

Finally, while many of the women preferred to inject themselves, as explained above, some preferred to inject in the company of a partner or friend to avoid certain risks, such as overdosing.

One participant explained how her friend helped her recover from overdose: *“I have overdosed twice because I’ve hit the wrong vein but a real overdose just once. I don’t remember anything really but [friend] injected me with salt water because he has experience with that; he has been using since he was twelve. When I woke up, I just remember he was talking to me and telling that I almost went to the other side* [dying] *and that I was turning purple. He injected me with salt and threw water at me and he had to hit me a couple of times*” (Ana, age 28 when interviewed, age 17 at initiation).

The women also explained events in which they helped to recover friends or other PWID from overdose.

*“I’ve had to bring people back to life. For example, yesterday I was here in San Ysidro* [on the U.S. side of the border] *and we went to the park and a friend of ours used more than what he should have and we told him before: ‘This one is strong, use less’ but this idiot didn’t listen to us and when he tried to get up, he fell and I started giving him CPR and thank god I was able to bring him back to life. We didn’t have anything* [naloxone] *there but I wasn’t going to let him die on me” (Sofia, age 39 when interviewed, age 23 at initiation).*

*“My boyfriend was overdosing once, and I injected him with naloxone. I injected him in the arm because he was already purple,”* another participant mentioned *(Isabel, age 20 when interviewed, age 16 at initiation).*

## Discussion

This qualitative study among WWID in Tijuana, Mexico, explored experiences ranging from injection initiation to current injection practices and identified several important findings that may further our understanding of risk and resilience among WWID in Tijuana. The injection experiences found among the women interviewed in this study build upon previously reported patterns of injection drug use among WWID and provide valuable insight into potential harm reduction interventions.

### Injection initiation

Experiences of injection initiation were shaped by the interplay of diverse interpersonal and structural factors at the micro- and macro-levels, explained by the risk environment framework [28]. Interpersonal relationships and gender dynamics were connected to each women’s unique experience of injection initiation through their micro-level social risk environment. Previous studies have found that being exposed to injection networks as well as having an intimate partner who is also an injector may influence injection initiation among women [2, 32–34]. Consistent with previous research, more than half of the participants in this study were initiated by a man who injects drugs, often in the context of an intimate relationship [2, 5, 10]. These results differ from some studies that concluded women tend to receive their first injection from another woman [11, 15], and suggest the persisting influence of gendered norms within Mexican society that permeate injection drug use patterns (i.e., patriarchal dynamics; men making choices for women by using either physical, sexual, or psychological power) [10]. Many of the women in this study discussed their dependence on their male partners for drug use and injection assistance, even beyond the initiation period. Current practices of injection drug were additionally influenced by perceptions of harm, such as wounds and overdose, within micro-level risk environments and shaped the participants decisions to inject with others or to help others to inject.

Additionally, political, economic, and physical contextual factors at the macro-level were emphasized as contributing to women’s injection initiation experiences. The context of Tijuana, a border city with high mobility of people and accessibility to drugs, has been noted by previous research as a key factor influencing initiation of injection drug use [20, 22, 23]. Some of the women connected their injection patterns to the hardships they faced due to medical issues, migration, or difficult family situations. The narratives illuminate the experience of drug dependence entrenched in these diverse contextual factors, indicating the need for interventions that not only seek to change individual-level practices, but also the macro-level structural factors and environments that produce individual experiences. As the findings demonstrate, in Tijuana, gender is seen to influence the choices available to women who inject drugs, thereby shaping their individual drug use practices [10].

### Self-injection

Importantly, this study develops understanding of the preference for self-injection from the perspective of WWID. While injection assistance and injection in the company of other PWID may be necessary at times to avoid harms, many of the women expressed that they would prefer to inject themselves, when possible. Relationships with other PWID were closely connected to perceived injection risks and the women’s efforts to minimize harm. Some women agreed that they only felt safe injecting with others who they had close relationships with and trusted, consistent with previous studies that have noted that women tend to buy and inject drugs with people they know and trust [10, 35]. However, many women mentioned their desire to inject alone, in private, due for safety concerns as well as fear of embarrassment or stigma associated with injection drug use [5]. These concerns underline the influence of the physical risk environment of injection drug use settings and characteristics, and previous exposure to violence or trauma. They described self-injection as their primary strategy for avoiding injection-related risks. The women agreed that injecting with others who they did not know or trust often led to additional risks. The perceived risks from allowing others to assist them with injection were greater than those that may result from injecting themselves.

In relation to the social and cultural context, the preference for self-injection highlights not only the perceived benefits from being able to inject safely and frequently, but also the benefit of establishing autonomy as a woman who injects drugs in Tijuana. The majority of women exhibited a “paradoxical autonomy,” explaining the dichotomy between dependence on MWID and individual control of one’s drug use trajectory [36]. The women’s survival strategies are seen to be linked to an attempt to conserve their autonomy and self-sufficiency throughout their drug use careers. Previous research indicated that women in Tijuana were restricted to injecting with other trusted women in their social networks in order to establish autonomy over their injecting practices within a context of police harassment against PWID [10]. This trend is supported by our findings of injecting others, where some participants reported helping other women to inject, particularly a partner or friend on a regular basis, illustrating the fact that trust and networks are key in this context and that the social and physical risk environments converge to influence protective strategies.

In our study setting, self-injection was discussed as an important strategy for reducing risks within the context of injection drug use in Tijuana; while injecting with and by other trusted PWID was used as a risk reduction strategy, injection with no assistance was perceived to carry the fewest overall risks. Strategically engaging in certain relationships and patterns of injection in order to minimize risks within the high-risk environment highlights the resilience of WWID in this specific context. The agency of WWID is produced as a form of self-defense, reinforced by experienced vulnerabilities, such as injection-related risks, intimate partner violence against women, and economic marginalization [26].

### Harm reduction interventions

The commonality of wounds, infections and other harms faced by WWID due to injection, specifically while learning to self-inject, highlight the need for accessible harm reduction interventions. Several studies have noted the lack of women-centered interventions globally and the need to implement targeted interventions due to the unique risks faced by WWID [9, 15, 16]. This qualitative study provides valuable information to inform harm reduction strategies from the perspectives of WWID. The results indicated that some women have the knowledge to self-inject but are unable to due to technical challenges with injecting or social pressure. As the risk environment framework portrays, multiple risk environment levels (i.e., social and physical risk environments) often intersect. Multiple risk environments at the micro- and macro-levels should therefore be considered and addressed by harm reduction interventions. The notable gap between the preference for self-injection and the tendency to continue to rely on others for injection indicates potential for interventions that support self-injection of women in order to reduce risks such as wounds, overdose, and theft.

Supervised consumption sites, with personnel trained in injection and responding to harms, such as wounds and overdose, would eliminate the need to rely on other PWID for injection and assist women in learning to self-inject safely [37, 38]. Additionally, connecting WWID to care for physical or mental health concerns is essential in building a population who is self-sufficient and in control of their injection drug use practices.

### Strengths and limitations

Although the small sample size and the qualitative nature of this study does not permit generalization of findings to a wider population, the testimonies provided by this study offer key contextual data regarding the experiences of WWID in Tijuana, Mexico, including their experiences of initiation and relying on others to inject as well as possible interventions. Whereas there have been several studies that note the divide in both research and risks faced by WWID in comparison with MWID, there have been few studies that closely qualitatively examine WWID experiences of initiation, transitioning, or protective strategies within a high-risk environment, such as the USA–Mexico border.

This study points to the need for more robust mixed-methods research to further detail more nuanced experiences that were revealed here, such as those of “street-hit doctors” and underage girls who are initiated into injection drug. Additional participatory research would also be beneficial in assessing the harm reduction interventions that might be most appropriate and feasible for the specific population of WWID in Tijuana, Mexico. Finally, larger studies in varied contexts are necessary to enhance these findings and generalize the experiences of WWID to additional contexts.

## Conclusions

WWID globally face an array of gender-specific risks shaped by diverse contextual factors and structural determinants. This exploratory, qualitative study enhanced our understanding of patterns of injection drug use among WWID, the risks they face, and protective strategies employed to mitigate those risks within the context of Tijuana, Mexico. The interviews detailed how micro-level interpersonal relationships with other PWID (e.g., sexual partner, friend, stranger) were closely linked to perceived risks faced by WWID. Many women relied on other PWID for drugs and injection assistance. However, the women’s relationships with other PWID, or lack thereof, were strategically linked to efforts to reduce risks while maintaining injection practices. The women in this study strongly expressed the importance of self-injection as a means of survival within the high-risk context of injection drug use in Tijuana. Self-injection was a safety strategy used to avoid the harms caused by relying on others and maintain autonomy and control over one’s injection practices. Together, these findings highlight the need for tailored interventions informed by harm reduction that consider the unique needs of WWID in Tijuana, Mexico.

## Data Availability

The datasets analyzed during the current study are available from the corresponding author under reasonable request.

## Abbreviations

FSWID: Female sex workers who inject drugs
MWID: Men who inject drugs
PWID: People who inject drugs
WWID: Women who inject drugs

## Footnotes

1 The preceding quotation is not an accurate description of the mechanism for HIV/AIDS transmission and is included solely to depict the emotions of the participant.
